# Bacterial Cyclodipeptides Target Signal Pathways Involved in Malignant Melanoma

**DOI:** 10.3389/fonc.2020.01111

**Published:** 2020-07-24

**Authors:** Mayra Xóchitl Durán-Maldonado, Laura Hernández-Padilla, Juan Carlos Gallardo-Pérez, Alma Laura Díaz-Pérez, Lorena Martínez-Alcantar, Homero Reyes De la Cruz, José Salud Rodríguez-Zavala, Gustavo Pacheco-Rodríguez, Joel Moss, Jesús Campos-García

**Affiliations:** ^1^Laboratorio de Biotecnología Microbiana, Instituto de Investigaciones Químico-Biológicas, Universidad Michoacana de San Nicolás de Hidalgo, Morelia, Mexico; ^2^Departamento de Bioquímica, Instituto Nacional de Cardiología, Mexico City, Mexico; ^3^Laboratorio de Control Traduccional, Instituto de Investigaciones Químico-Biológicas, Universidad Michoacana de San Nicolás de Hidalgo, Morelia, Mexico; ^4^Pulmonary Branch, National Heart, Lung, and Blood Institute, National Institutes of Health, Bethesda, MD, United States

**Keywords:** antitumor activity, cyclodipeptides, tumorigenesis, melanoma, cell proliferation, apoptosis, epithelial–mesenchymal transition

## Abstract

Melanoma is an aggressive cancer that utilizes multiple signaling pathways, including those that involve oncogenes, proto-oncogenes, and tumor suppressors. It has been suggested that melanoma formation requires cross-talk of the PI3K/Akt/mTOR and Ras-ERK pathways. This pathway cross-talk has been associated with aggressiveness, drug resistance, and metastasis; thus, simultaneous targeting of components of the different pathways involved in melanoma may aid in therapy. We have previously reported that bacterial cyclodipeptides (CDPs) are cytotoxic to HeLa cells and inhibit Akt phosphorylation. Here, we show that CDPs decreased melanoma size and tumor formation in a subcutaneous xenografted mouse melanoma model. In fact, CDPs accelerated death of B16-F0 murine melanoma cells. In mice, antitumor effect was improved by treatment with CDPs using cyclodextrins as drug vehicle. In tumors, CDPs caused nuclear fragmentation and changed the expression of the Bcl-2 and Ki67 apoptotic markers and promoted restoration of hyperactivation of the PI3K/Akt/mTOR pathway. Additionally, elements of several signaling pathways such as the Ras-ERK, PI3K/JNK/PKA, p27Kip1/CDK1/survivin, MAPK, HIF-1, epithelial–mesenchymal transition, and cancer stem cell pathways were also modified by treatment of xenografted melanoma mice with CDPs. The findings indicate that the multiple signaling pathways implicated in aggressiveness of the murine B16-F0 melanoma line are targeted by the bacterial CDPs. Molecular modeling of CDPs with protein kinases involved in neoplastic processes suggested that these compounds could indeed interact with the active site of the enzymes. The results suggest that CDPs may be considered as potential antineoplastic drugs, interfering with multiple pathways involved in tumor formation and progression.

## Introduction

Communication between the microbiome and host cells plays important roles in health and disease (1). Microbes produce metabolites capable of affecting cellular signaling pathways and thus could become potent therapeutics (2). For instance, structurally diverse cyclodipeptides (CDPs) of bacterial (3, 4) and with less efficiency of synthetic origin (5) are cytotoxic to human cancer cell lines. In fact, we have shown that CDPs [cyclo(L-Pro-L-Tyr), cyclo(L-Pro-L-Val), and cyclo(L-Pro-L-Phe)] from *Pseudomonas aeruginosa* PAO1 promoted apoptosis and cell death of human cervical (HeLa) and colorectal adenocarcinoma (CaCo-2) cells, whereas normal human lung fibroblasts were insensitive (6). The molecular mechanisms used by CDPs to trigger cytotoxicity, leading to death of cancer cells, appear to involve microtubule polymerization (7) and caspase-3 activation (3, 6).

Cancer results from dysfunction of fundamental cellular processes. In fact, pathways involving oncogenes and tumor suppressors are frequently involved in cancer development and progression (8, 9). Interestingly, the mechanistic target of rapamycin (mTOR) serine/threonine kinase is a master regulator that participates in two complexes (mTORC1 and mTORC2), and its dysregulation has been implicated in cancer. mTORC1 has been implicated in cellular processes, such as, energy metabolism, proliferation, tumorigenesis, and autophagy, whereas the mTORC2 complex is involved in actin cytoskeleton reorganization and survival (10). mTORC1 activity is frequently up-regulated in cancer, particularly following increased oncogenic activation of phosphoinositide 3-kinase (PI3K) signaling or inactivation of the lipid phosphatase PTEN (phosphatase and tensin homolog) (9, 11).

Multiple biomarkers characterize a neoplasm/cancer and metastasis (9, 10, 12), which in many cases is initiated by cancer stem cells (CSC) and may involve epithelial–mesenchymal transition (EMT). Epithelial–mesenchymal transition has been associated with action of N-cadherin, a membrane protein involved in cell attachment, which is up-regulated during metastasis and invasion, and promotes tumorigenesis. Additionally, direct interaction of N-cadherin with PI3K may enable activation of the PKB/Akt pathway, suggesting that it could be a therapeutic target in cancer (13). N-cadherin can also promote cell survival, migration/invasion, and the EMT process by direct cross-talk with other signaling pathways, [e.g., nuclear factor κB (NFκB)–mediated, mitogen-activated protein kinase (MAPK), receptor tyrosine kinase (RTK), Ras homolog family member A small GTPase protein (RhoA GTPase), PI3K (14)]. Otherwise, EMT is a crucial regulatory pathway with links to embryogenesis and cancer development.

In melanoma, multiple signaling pathways are dysregulated, involving oncogenes and tumor suppressors (i.e., PI3K/AKT/mTOR, MAPK, RAS/MEK/ERK, BRAF, and CDK); the multiple dysregulation of these signaling pathways favors tumor invasiveness, progression, drug resistance, and recurrence. Current therapeutic procedures for melanoma include chemotherapy, immunotherapy, biochemotherapy, and gene therapy (15, 16). However, participation of multiple signaling pathways in melanoma pathology complicates its treatment. Then, the elucidation of the involvement of EMT and CSC pathways in melanoma invasiveness, drug resistance, and recurrence is crucial. The main goal of this study was to evaluate the effects of CDPs on a xenografted melanoma tumor model and elucidate the molecular mechanisms involved in CDP action. We observed that CDPs killed melanoma cells and decreased tumor burden. During melanoma development, multiple cell-signaling pathways were targeted and restored by bacterial CDPs, suggesting that these molecules have the potential for use as antiproliferative drugs.

## Materials and Methods

### Chemicals and Reagents

Dulbecco modified Eagle medium (DMEM), fetal bovine serum (FBS), 3-(4,5-dimethylthiazol-2-yl)-2,5-diphenyltetrazolium bromide (MTT), and β-cyclodextrin (β-cyclodextrin hydrate were purchased from Sigma-Aldrich Co., St. Louis, MO, USA). Alexa Fluor 488 annexin V and the propidium iodine (PI)/dead cell apoptosis kits were from Invitrogen Life Technologies, Carlsbad, CA, USA. Cyclodipeptides were obtained from *P. aeruginosa* PAO1 and characterized as previously described (17, 18).

### Cell Culture

Mouse B16-F0 melanoma cells line was obtained from the American Type Culture Collection (ATCC, Manassas, VA, USA). Cells were cultured in complete media (CM) [DMEM supplemented with 10% (vol/vol) FBS, 100 U/mL of penicillin, 40 μg/mL of streptomycin, and 1 μg/mL of amphotericin B (Sigma-Aldrich Co., St. Louis, MO, USA)]. Cell culture media were changed twice a week and maintained at 37°C under 80% humidity and incubated in an atmosphere of 5% CO_2_. Following trypsinization, cells were grown to confluency; cells were counted using a hemocytometer chamber.

### Cell Viability, Necrosis, and Apoptosis Assays

Cell viability was determined colorimetrically with MTT. Briefly, cells were seeded in 96-well flat-bottomed plates at a density of 3 × 10^4^ cells per well in 200 μL of CM medium and incubated by 24 h at 37°C with 5% CO_2_ as described above. Then, cell culture media were removed and replaced with serum-free DMEM. Following incubation in DMEM with FBS for 24 h, cells were incubated in the presence or absence of the indicated amounts of CDPs for 24 h at 37°C with 5% CO_2_. To determine cell viability, MTT, 50 mg/mL in phosphate-buffered saline (PBS), was added to each well and incubated for 4 h at 37°C. Finally, 100 μL of 2-propanol/1 M HCl (19:1 vol/vol) was added to dissolve formazan crystals, and absorbance was measured at 595 nm using a microplate reader (BioTek Instruments, Winooski, VT, USA).

To quantify necrosis and apoptosis, cell cultures were incubated with DMEM with FBS for 12 h prior to treatment with CDPs. Dimethyl sulfoxide (DMSO) was used as a control at the same concentration used to dissolve the CDPs. Following incubation, cells were collected by centrifugation at 2,000 g for 10 min. The pellet was suspended in 20 μL and incubated with annexin V and propidium iodide (PI) (Dead Cell Apoptosis Kit; Molecular Probes, Invitrogen Life Technologies, Carlsbad, CA, USA). Fluorescence was immediately quantified by fluorescence-activated cell sorting (FACS) using an Accuri-C6 Flow Cytometer (BD Biosciences, San Jose, CA, USA). The percentages of fluorescent cells (PFC) and median fluorescence intensity were determined from histograms of the fluorescence emission in the plots, labeled as PFC or as relative fluorescence units. For apoptosis and necrosis assays, annexin V fluorescence was measured with fluorescence channel FL1 at 495/519 nm and for PI in FL2 channel at 535/617 nm. At least 20,000 cellular events were used for calculations.

### Subcutaneous Xenografted Melanoma Mouse Model

All the experiments using mice complied with standard guidelines for the welfare of animals with experimental neoplasia in accordance with the recommendations of the Mexican Official Regulations for the Use and Care of Animals (NOM 062-ZOO-1999; Ministry of Agriculture, Mexico). This research was also approved by the Institutional Committee for Use of Animals of the Universidad Michoacana de San Nicolás de Hidalgo. Male C57BL/6 mice between the ages of 4 and 5 weeks were purchased from Envigo RMS S.A. (Mexico City, Mexico). Animals were hosted in facilities for 2 weeks before experiments were started; afterward, the xenograft melanoma procedure was conducted as described below. The mice were housed separately in ventilated cages under a controlled light cycle (12-h light/12-h dark) at room temperature (22–26°C) and were fed with a standard rodent diet and water *ad libitum* in accordance with NOM 062-ZOO-1999; Ministry of Agriculture, Mexico.

Melanoma cells were injected subcutaneously in mice at the right flank with 2 × 10^5^ B16-F0 murine melanoma cells. Tumor size was measured every 2 days. Two bisecting diameters of each tumor were measured with calipers. The volume was calculated using the formula (0.4) (ab^2^), with “a” being the larger diameter and “b” being the smaller diameter (19). All mice injected with melanoma cells developed tumors of 20–50 mm^3^ 8 days from the day of injection. The CDPs were administered at a dose of 0.1 mg/g of mice weight, which was determined assuming that the corporal volume of mice is ~50 cm^2^. A concentration of 50 μg/mL (50% apoptotic cells in the assay) was used, rendering 2.5 mg per mouse per dose. In addition, two additional doses of CDPs were tested in a pilot study, with CDP treatment at 10, 50, and 200 μg/mL of CDPs. The results showed similar antitumorigenic effect at 50 and 200 μg/mL of CDPs, but no significant results with 10 μg/mL of CDPs; thus, we decided to use 50 μg/mL of CDPs for this study. A 50 mg/mL stock solution of CDPs was prepared by dissolving the compounds in sterile saline solution containing DMSO (0.1 %), or β-cyclodextrin (50 mg/mL). All liquid treatments of the mouse model were injected in the caudal vein as authorized by NOM 062-ZOO-1999; Ministry of Agriculture, Mexico. Seven different groups of six mice each were randomly assigned: (1) control, healthy mice were injected with 50 μL of sterile saline solution with DMSO (0.1%); (2) control + CDPs, healthy mouse group injected with 50 μL of CDP stock solution; (3) tumor (T), mice with melanoma tumors were injected with 50 μL of sterile saline solution with DMSO (0.1%); (4) T + CDPs-t0, mice with melanoma cell injections were treated from the beginning with a weekly injection with 50 μL of CDP stock solution (three total doses); (5) T + CDPs-t8, mice with melanoma tumors (with volume of ~20–50 mm^3^ after 8 days) were injected weekly with 50 μL of CDP stock solution (two total doses); (6) T + CDPs-cdxt0, mice as in group 4 were injected weekly with 50 μL of CDP stock solution in β-cyclodextrins (50 mg/mL, three total doses); and (7) T + CDPs-cdxt8 mice as in group 5 were treated weekly with 50 μL of CDP stock solution in β-cyclodextrin (50 mg/mL, two total doses). The mice were examined every 2 days and weighed, and tumor size was measured until they were euthanized (20 days) as authorized by the Institutional Committee for Use of Animal of the Universidad Michoacana de San Nicolás de Hidalgo in accord to the NOM 062-ZOO-1999; Ministry of Agriculture, Mexico. Then, organs were removed, tumor area was determined, and dissected tumors and organs were stored at −80°C prior to histopathological studies. Mice that died of causes unrelated to the neoplasm and CDP treatment were not considered in the analysis.

### Blood Parameters Evaluation

Blood was obtained by cardiac puncture prior to euthanizing as recommended (NOM 062-ZOO-1999; Ministry of Agriculture, Mexico) and collected in tubes containing heparin. Blood was centrifuged at 3,500 g for 10 min to obtain plasma. Hematocrit was measured by centrifugation (10,000 g for 5 min) using a hematocrit capillary tube. Hemoglobin was determined as follows: Hb = (hematocrit value) (3.3 factor). Plasma was utilized to measure lactate dehydrogenase (LDH), aspartate aminotransferase (AST), and alanine aminotransferase (ALT) activities using the Dry Chemical Analyzer Vitros 350 (Ortho Clinical Diagnostics, Wooburn Green, Buckinghamshire, UK).

### Histological Analysis of Tumor Tissue

Tumors and tissues from mice were excised and fixed in 10% neutral-buffered formaldehyde solution and embedded in paraffin. Tissue sections (0.2 mm × 10 μm) were obtained using a Cryostat (Leica CM1850, Leica Biosystems Inc., Buffalo Grove, IL, USA) and stained with hematoxylin-eosin. For immunohistochemical analysis, formalin-fixed tissue sections were dehydrated in a sucrose gradient (10–30%) in PBS buffer for 48 h each. Slides were treated with 10 mM citrate buffer at 60°C, permeabilized with PBS-T buffer, and incubated with H_2_O_2_, followed by incubation with 5% horse serum in PBS buffer for 2 h at 25°C. Then, tissue sections were incubated with anti-mouse Bcl-2 and anti-mouse-Ki67 antibodies (Santa Cruz Biotechnology, Santa Cruz, CA, USA) with a ratio of 1:100 in PBS buffer containing 0.2% horse serum for 24 h at 25°C. The slides were washed three times with PBST buffer, followed by incubation with the secondary antibody (1:500) in PBS [anti-mouse immunoglobulin G (IgG) biotinylated; Vector Labs] for 1 h at 25°C. Antibody reaction was developed using the Vectastain Elite ABC horseradish peroxidase (HRP) kit (Vector Labs, Inc., Burlingame, CA, USA). In case of frozen tissue, tissue sections of 7 μm were obtained with a cryostat Hyrax C25, Carl Zeiss, Gottingen, Germany, at −20°C. Images were acquired with a contrast phase inverted-fluorescence microscope (Leica DM 3,000 equipped with a digital CDD, Leica Biosystems Inc., Buffalo Grove, IL, USA). In addition, tumor cells treated with rhodamine 123 (Sigma-Aldrich Co. St. Louis, MO, USA) were observed directly using a confocal microscope (Olympus FV1000, Center Valley, PA, USA); the emission signal of fluorescence was monitored at 533 to 563 nm for rhodamine 123 probe.

### Antibody Array Assay

Melanoma tissue from three to six mice (~3 g) were cut into small pieces on ice and homogenized by sonication in 300 μL of phosphorylation buffer (HEPES 50 mM pH 7.6 containing sodium-pyrophosphate 50 mM, sodium orthovanadate 1 mM, sodium molybdate 1 mM, EDTA 20 mM, EGTA 20 mM, benzamidine 1 mM, NaF 20 mM, PMSF 0.2 mM, β-glycerophosphate 80 mM, mannitol 200 mM, and protease inhibitor cocktails 1 μL/mL). Then, we used three cycles of sonication at low intensity (20 kHz, 5 W) for 30 s each at 4°C with 5 min of resting between sonication cycles (Hielscher-LS24 Ultrasound Technology, Ringwood, NJ, USA). Cell-free protein extracts were obtained by centrifugation (7,500 g, 4°C for 15 min). Protein concentration was determined using the Bradford reagent (BioRad, Hercules, CA, USA) and 30 μg of total protein were added to each well with glass slides of antibody array kit (PathScan Cancer Phenotype Antibody Array Kit #14,821 and PathScan Intracellular Signaling Array Kit #7,323; Cell Signaling Technology, Danvers, MA, USA). The array glass slides were incubated overnight at 4°C on an orbital shaker. Following immunoreactions and washes, slides were incubated with a biotinylated-antibody cocktail and HRP-linked streptavidin for 1 h at room temperature. To detect immunoreactivity, LumiGlo®/Peroxide reagent was added and the image immediately captured using a digital imaging chemiluminescent system, ChemiDoc™ MP System (BioRad, Hercules, CA, USA). Determination of spot intensity from the microarray was carried out by densitometry analysis using ImageJ software (NIH Image).

### Western Blot Analysis

Proteins from tumor extracts were separated under denaturing conditions using polyacrylamide gel electrophoresis at 10–12% [sodium dodecyl sulfate (SDS)]. Thirty micrograms of protein extracts were typically loaded per lane. Protein mixtures were mixed with 10 μL of denaturing buffer (Tris-HCl 0.06 M, pH 6.8, 5% glycerol, 4% SDS, 4% β-mercaptoethanol, and 0.0025% bromophenol blue) and incubated for 5 min at 95°C. Gels were stained with Coomassie blue and proteins from replicate gels transferred to polyvinylidene difluoride (PVDF; Millipore, Billerica, MA, USA) membranes for immunodetection assays. Briefly, PVDF membranes were incubated with TBS-T (Tris-HCL 10 mM; NaCl 0.9%; tween-20 0.1%, dry milk 5%, pH 7.8). Polyvinylidene difluoride membranes were cut according to range of molecular weight markers and incubated with the indicated antibodies at the concentration suggested by the manufacturer: anti-CD44, anti-Oct3/4, anti-C-Myc, anti-Ras, anti-SNAIL, anti-MMP-1, anti-E-Cad, anti-vimentin, anti-cytokeratin 1 (CK-1), anti-α-tubulin, anti-Akt (C-20), anti-Akt-phosphorylated 1/2/3 (S-473), anti-mTOR, anti-phosphorylated-mTOR-(S2448), anti-β-actin, and anti-α-tubulin (all from Santa Cruz Biotechnology, Santa Cruz, CA, USA) antibodies. Following 12 h of incubation (4°C) for the primary antibody, membranes were washed and incubated with goat anti-rabbit IgG HRP-conjugate (1:10,000, BioRad, Hercules, CA, USA), in blocking medium for 4 h at 4°C; the membranes were washed twice with TBS-T buffer and developed using hydrogen peroxide and Supersignal West Pico Luminol (Pierce; Thermo Fisher Scientific, Waltham, MA, USA). Then images were captured using a ChemiDoc™ MP System (BioRad, Hercules, CA, USA). Assays were conducted at least three times, and representative images are shown. Band intensities in gel images or films were quantified using the ImageJ software (NIH Image).

### Docking Analysis

Data of the protein structure of mice, rat, and human were obtained from the protein data bank [accession no. AKT (3CQU), HIPK2 (6P5S), AMPK (5UFU), MET (3QTI), JNK (2G01), CD44 (2JCR), and HIF-1α (5JWP)]. The three-dimensional models of CDPs used in the study were obtained from https://pubchem.ncbi.nlm.nih.gov/compound/ and as previously described (18). Docking analysis was carried out using the software Autodock 4.2.5.1 (available at http://autodock.scripps.edu/). After docking, 100 conformations for each compound were obtained and then clustered for analysis using ADT 1.5.2 software. The conformations selected were within the most represented cluster and corresponded to those showing the lowest values of binding energy and *Ki*. Model analyses and figure drawing were carried out with PYMOL 2.1.0 (The PyMOL Molecular Graphics System, version 2.1.0; Schrödinger, LLC, New York, NY, USA; https://sourceforge.net/p/pymol).

### Statistical Analysis

For correlation analysis, data obtained of antibody arrays and Western blots were analyzed by correlation analysis utilized as response variables (treatments) vs. data of signal intensity for each antibody (cases) using the STATISTICA software (Data Analysis Software System 8.0; Stat Soft Inc., Tulsa, OK, USA). Other data were statistically analyzed using GraphPad Prism 6.0 software (GraphPad Software, San Diego, CA, USA).

## Results

### CDPs From *P. aeruginosa* PAO1 Induce Apoptosis of Murine B16-F0 Melanoma Cells

**Figure 1:**
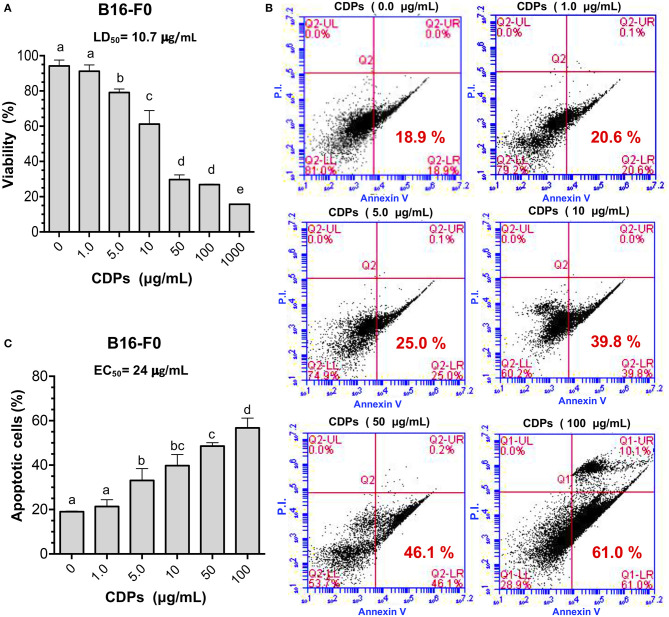
Effects of CDPs on viability and apoptosis of B16 murine melanoma cells. B16-F0 melanoma cells were incubated with CDPs to determine cell viability and apoptosis as described in Materials and Methods. **(A)** Determination of viability of B16-F0 melanoma cells treated with different concentrations of CDPs from *P. aeruginosa* PAO1 by MTT method; LD_50_ of CDPs is shown. **(B,C)** Measurement of apoptosis using annexin V binding and necrosis by propidium iodide (PI) were conducted by flow cytometry using different concentrations of CDPs. **(B)** Representative flow cytometry dot plots show the annexin V and PI cells percentage of CDPs-treated B16-F0 cells. **(C)** Determination of apoptosis induction by CDPs and EC_50_ is shown. Bars represent means ± SE of three independent assays, *n* = 3. One-way analysis of variance (ANOVA) was carried out, with Tukey *post-hoc* test; statistical significance (*P* < 0.05) of differences between treatments is indicated with lowercase letters.

First, we examined the effect of these compounds on the B16-F0 melanoma line in culture, finding that CDPs decreased viability of B16-F0 cells in a dose-dependent manner. Cell cultures showed 75% dead cells with CDPs at 50 μg/mL after 12 h, rendering an LD_50_ of 10.7 μg/mL (Figure 1A); remarkably, the LD_50_ data indicated that CDPs were ~5-fold more bioactive in inducing cell death in the B16-F0 murine melanoma line than in the human HeLa line described previously (6). To further support the CDP effect on the B16-F0 melanoma line, we used annexin V binding to determine apoptotic and PI to determine necrotic cells by FACS analysis (Figures 1B,C). Whereas, <20% of cells were stained with annexin V in cells incubated with vehicle, ~60% of cells treated with CDPs (50 μg/mL at 4 h) became apoptotic (Figure 1C). The EC_50_ of apoptosis induction was ~24 μg/mL at 4 h of treatment (Figure 1B). These data indicated that CDPs from *P. aeruginosa* PAO1 were cytotoxic, inducing apoptosis in the B16-F0 melanoma cell line.

### Effect of CDPs on Xenografted Melanoma Murine Tumors

**Figure 2:**
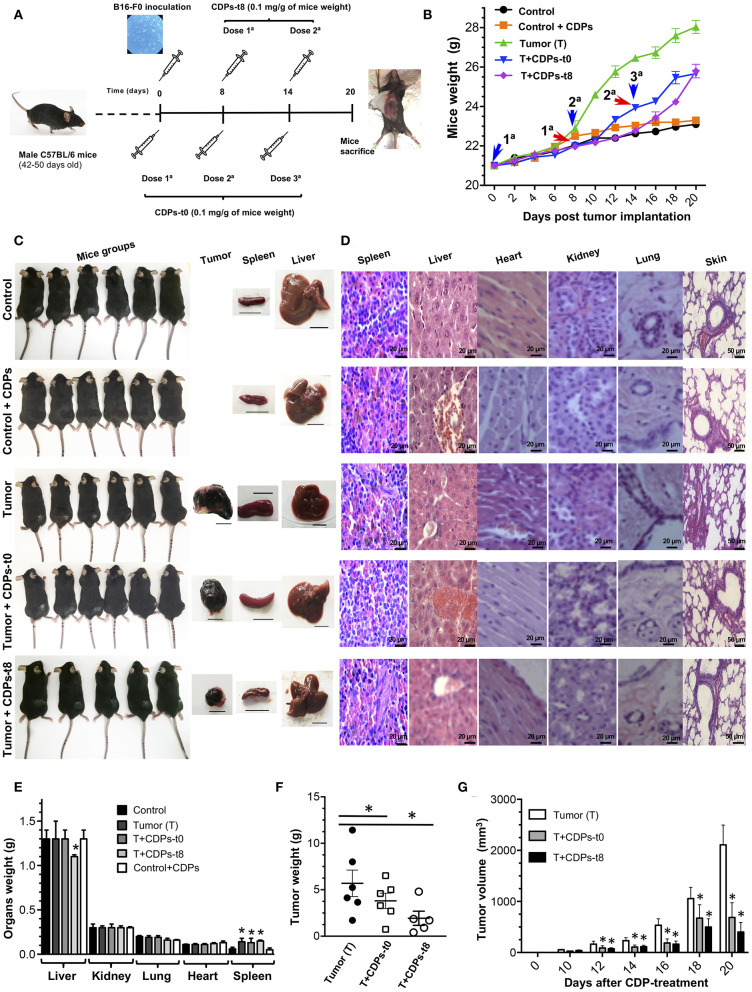
Antitumor effect of CDPs on xenografted tumor melanoma B16-F0 cells in mice. **(A)** Experimental design showing tumor implantation and CDP treatments. Mice of 6 weeks of age were weighed, melanoma was implanted subcutaneously, and treatments were started. Mice (*n* = 6 per group) were treated as indicated. After 20 days, mice were euthanized, organs dissected, and diverse parameters were determined as described in Materials and Methods. **(B)** Determination of mouse weight over 20 days' period. Arrowheads indicate the CDP administration time and dose number. **(C)** Photograph of mouse groups at the 20th day of experimental procedure before euthanizing. Representative photographs of tumor, spleen, and liver from each mouse group are shown. Scale bar was assigned adjusting the scale with the original ruler corresponding to 10 mm. **(D)** Representative hematoxylin-eosin staining of tissue from spleen, liver, heart, kidney, lung, and skin of the mouse groups. Scale bar in μm is shown. Determination of weight of mouse organs after 20 days **(E)**, tumor weight **(F)**, and tumor volume **(G)**. Bars represent the means ± SE of one experiment, *n* = 6. One-way ANOVA with Bonferroni *post-hoc* test was used to compare treatments with the untreated group (control) or with tumor group (T); significant differences (*P* < 0.05) are indicated with asterisk (*).

To evaluate the antitumorigenic potential of a mixture of bacterial CDPs, we used a subcutaneous xenografted melanoma mouse model (20). Tumors were induced by subcutaneous injection of B16-F0 melanoma cells on male C57BL/6 mice of 10 weeks of age. To determine the effect of CDPs on melanoma development, different mouse groups were tested as described in *Materials and Methods*. Typically, after 8 days, the tumors reached a volume average of 20–50 mm^3^. A group of mice was treated with CDP injection from the beginning of cells implantation (T + CDPs-t0) (Figure 2A). Another mouse group was treated after the eighth day of melanoma cells implantation [T + CDPs-t8]. To monitor tumor growth, mice were weighed every 2 days for 20 days (Figure 2B). The body weight of mice belonging to the tumor (T) group showed a significant increment in weight. Body weight of mice in both the T + CDPs-t0 and T + CDPs-t8 groups were lower than the T group without CDP treatment, suggesting that the weight increment found in the T group could be related to tumor development. Healthy mice treated with CDPs did not show changes in body weight compared to the control group. All mice survived for 20 days, were humanely euthanized, and tumors and organs removed and weighed (Figure 2C). There were no significant differences in weights of kidney, lung, and heart organs; however, a 2-fold increase in the weight and volume of the spleen of the T mouse group was observed (Figures 2C–E). A decreased liver weight was also observed in the T + CDPs-t8 group (Figure 2E).

Data show that CDP treatment caused a significant decrease in mass and volume of tumors (Figures 2F,G). We found that the average weight of the tumors in mice without treatment (T) was ~5.5 g, with a tumor volume of ~2,100 mm^3^, whereas the tumors from the CDP-treated mouse groups injected immediately with CDPs (T + CDPs-t0), and after the eighth day of melanoma cells implantation [T + CDPs-t8], showed on average ~4 and ~2 g, with volumes of ~70 and ~30 mm^3^, respectively, (Figures 2F,G). Thus, CDPs decreased size and weight of xenografted tumors formed by B16-F0 melanoma cells in C57BL/6 mice.

Histopathological studies showed no apparent modification of cell size, morphology, and cellular structures from spleen, liver, heart, kidney, lung, and skin in the mice groups (Figure 2D). In addition, tissues from the healthy CDP-treated control mouse group were normal; thus, CDP treatments appear to be safe for mice.

**Figure 3:**
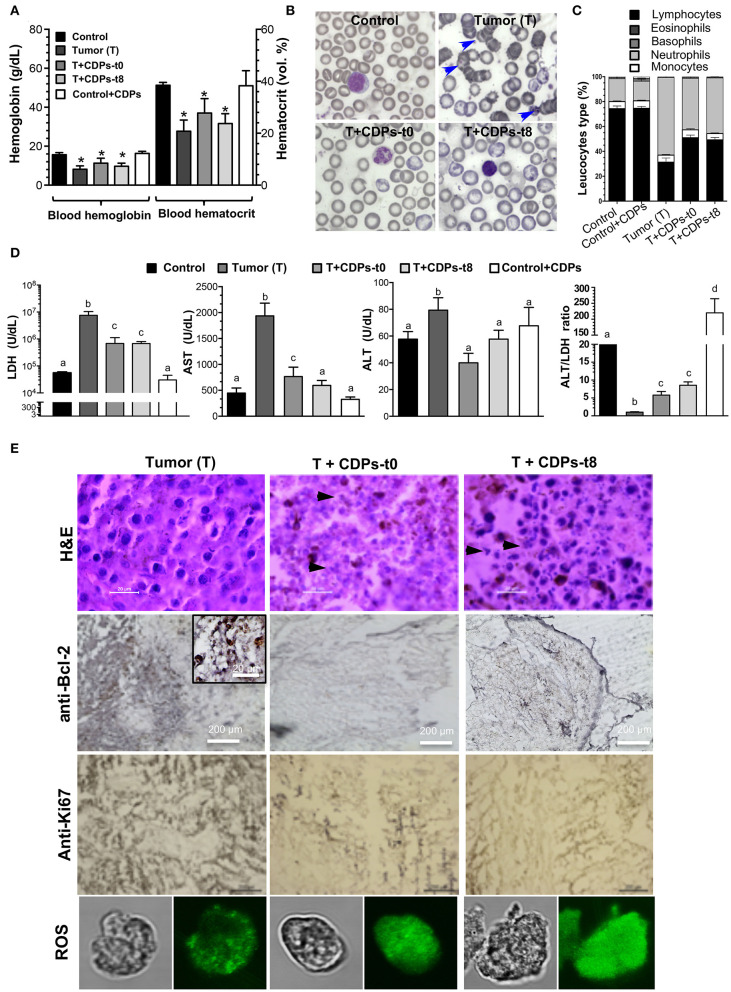
Determination of tumor markers in xenografted tumor melanoma mice treated with CDPs. **(A)** Determination of hemoglobin and hematocrit in the blood of the different mouse groups at 20^th^ day of experimental procedure before euthanizing. **(B)** Morphology of blood cells (echinocytes are indicated with arrowheads). **(C)** Leukocyte differential. **(D)** Determination of lactate dehydrogenase (LDH), aspartate aminotransferase (AST), and alanine aminotransferase (ALT) activities, and the ALT/LDH ratio. Bars represent the means ± SE, *n* = 6. One-way ANOVA with Bonferroni *post-hoc* test was used to compare treatments with the untreated group (control). Significant differences (*P* < 0.05) are indicated with asterisk (*) or with lower-case letters. **(E)** Histological analysis of tissue from melanoma tumors by hematoxylin-eosin (H&E) staining, anti-Bcl-2, anti-Ki67 immunoreactivity, and confocal images of tumor cells stained with rhodamine 123 (ROS probe). Scale bar is shown. Arrowheads indicate nucleus fragmentation. Control, healthy mice; control + CDPs, healthy mice treated with CDPs; Tumor (T), mice with tumor implantation without treatment; T + CDPs-t0, mice with tumor implantation and immediately treated after injection; T + CDPs-t8, mice with tumor implantation with CDP treatment at the eighth day of melanoma implantation.

Hemoglobin and hematocrit were significantly diminished in the T group (Figure 3A). The control mouse group treated with CDPs showed no differences in any of the hematological parameters. Erythrocytes from the T mice group showed characteristic evidence of echinocytes (frequently found in patients with liver disease; Figure 3B). We found that the T mouse group had a reduction in the proportion of lymphocytes accompanied by an increment of neutrophils; these effects were less pronounced in mouse groups treated with CDPs (Figure 3C).

We also determined the activity of cell damage marker enzymes LDH, AST, and ALT. The T mouse group showed a significant increment in both LDH and AST enzymatic activities (Figure 3D), which were diminished in the tumor-induced mice treated with CDPs (T + CDPs-t0 and T + CDPs-t8). The treatment with CDPs in the healthy CDP-treated control mouse group did not alter LDH and AST levels as shown in the control mouse group. Alanine aminotransferase activity did not show significant differences between the mouse groups, except for the T mouse group. Nevertheless, the ALT/LDH ratio showed significant difference with tumor development and CDP treatment. An increased ALT/LDH ratio (~10-fold) was observed in the healthy mouse group administered with CDPs (control + CDPs), whereas a lower value of this ratio was observed in the tumor group without CDP treatment (Figure 3D).

Histopathological examination showed strong nuclear fragmentation in cells of tumor tissue from mice treated with CDPs (T + CDPs-t0 and T + CDPs-t8); this effect was not observed in the tissues from the untreated (T) mice group (Figure 3E). Bcl-2 and Ki67 tumor markers were significantly diminished in the mouse groups that were CDP treated (Figure 3E). Additionally, determination of reactive oxygen species (ROS) on cells dissected from tumor tissues using the rhodamine 123 probe was carried out. Confocal microscopic images showed an exacerbated generation of ROS in the cells from tumors of mice treated with CDPs, as well as a loss of cell morphology (Figure 3E), indicating an apoptotic and necrotic status on tumor tissues, induced by CDP treatment.

### Cyclodextrins Favor the Antitumor Effect of the CDPs in Xenografted Mouse Melanoma

**Figure 4:**
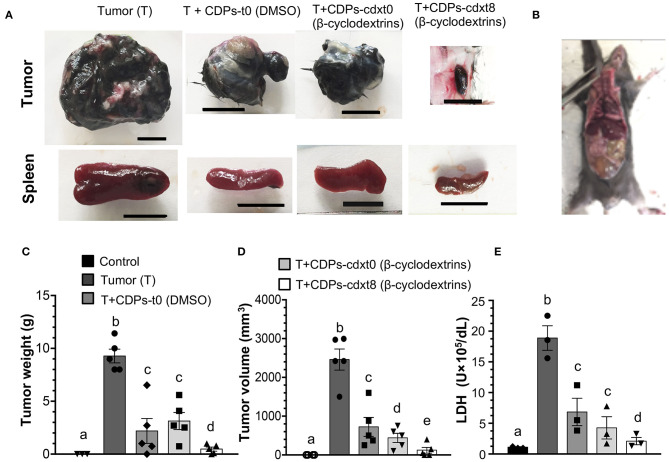
Effects of CDPs suspended in cyclodextrins on xenografted tumor melanoma B16-F0 cells in mice. Mice from the different groups were treated with/without CDPs using as drug vehicle a suspension of cyclodextrins in saline solution. After 20 days of treatment, mice were euthanized, tumors weighed, and LDH activity determined as described in Materials and Methods. **(A)** Representative photographs of tumors and spleens from mouse groups treated with CDPs–cyclodextrins (CDPs-cdx). Scale bar was assigned corresponding to 10 mm. **(B)** Xenografted melanoma mouse that after CDPs–cyclodextrins treatment did not show any tumors. **(C,D)** Tumor weight and volume determination. **(E)** LDH activity in mouse serum. DMSO indicates that CDPs were dissolved in saline solution with DMSO (0.1%), (β-cyclodextrins) indicates that CDPs were dissolved in saline solution with β-cyclodextrins (50 mg/mL). Bars represent means ± SE, mouse groups with *n* = 3–6. One-way ANOVA with Bonferroni *post-hoc* test was used to compare treatments to the untreated group (control). Significant differences (*P* < 0.05) are indicated with lowercase letters. Control, healthy mice; Tumor (T), mice with tumor implantation without treatment; T + CDPs-t0 (DMSO), mice with tumor implantation with CDPs (DMSO-dissolved) and immediately treated; T + CDPs-cdx-t0 and T + CDPs-cdx-t8, mice with tumor implantation with CDP treatment (β-cyclodextrins–dissolved) immediately after melanoma cell injection and at the eighth day of melanoma implantation, respectively.

Because DMSO was the vehicle used to solubilize CDPs, we wondered if the beneficial effect observed on mice with melanoma implantation could be improved by another drug vehicle; we replaced DMSO with a β-cyclodextrin suspension. The CDP–cyclodextrin suspension was administered to the mouse groups with xenografted tumors and compared with treatments where CDPs were dissolved in DMSO. Tumors of the mouse group without treatment (T) showed an average weight of ~9 g with a volume of ~2,200 mm^3^. The average weight and volume of tumors from mice treated with CDP-dissolved in DMSO were ~2.5 g and ~900 mm^3^, respectively, (Figures 4A,C,D). The mouse group treated with the CDP–cyclodextrin suspension administered at the eighth day after melanoma cell injection (T + CDPs-cdxt8) showed an average tumor weight of ≤ 0.3 g and a volume of ≤ 50 mm^3^; interestingly, in some mice (20%), the tumor was undetectable (Figure 4B). The mouse group treated with CDP–cyclodextrins suspension injected immediately (T + CDPs-cdxt0) showed similar tumor weight with DMSO–CDP treatments, but it showed a significant decrease in tumor volume (Figures 4C,D). Additionally, the cyclodextrins–CDP treatments significantly decreased spleen size and LDH activity compared to those of DMSO–CDPs treated and untreated mouse groups (Figures 4A,E). The lowest LDH activity was observed in the T mouse group treated with CDP–cyclodextrins at the eighth day after tumor implantation (T + CDPs-cdxt8). These results indicate that CDPs were more efficient when they were injected with cyclodextrins suspension compared to DMSO.

### Modulation of Signaling Pathways by CDPs Treatment in Xenografted Mouse Melanoma

**Figure 5:**
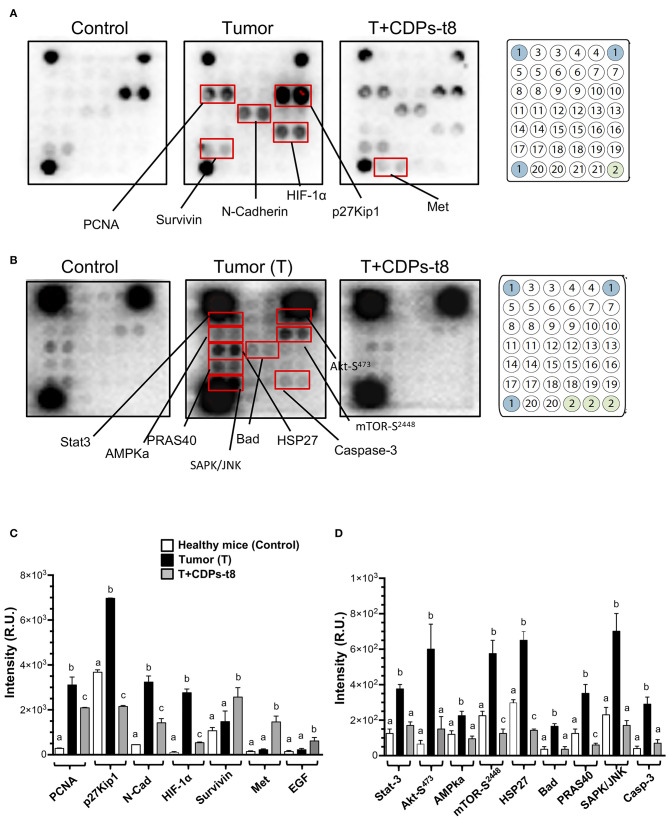
Proteomic analysis of CDPs effect on xenografted tumor melanoma B16-F0 cells in mice. Tumors or healthy skin were homogenized to obtain the tissue lysates used for Western blot assays as described in Materials and Methods. **(A)** Representative images correspond to antibody microarrays (PathScan Cancer Phenotype Antibody Array). The arrays contained the following antibodies: (1) positive control, (2) negative control, (3) CD31, (4) EpCAM, (5) vimentin, (6) CD44, (7) CD45, (8) PCNA, (9) Ki-67, (10) p27Kip1, (11) E-cadherin, (12) N-cadherin, (13) VE-cadherin, (14) MUC1, (15) Rb Ser807/811, (16) HIF-1α, (17) survivin, (18) P53, (19) HER2/ErbB2, (20) Met, (21) EGF. **(B)** Representative images correspond to antibody micro-arrays (PathScan Intracellular Signaling Array). The arrays contained the following antibodies: (1) positive control, (2) negative control, (3) ERK1/2-Thr202/Tyr204, (4) Stat1-Tyr701, (5) Stat3-Tyr705, (6) Akt-Thr308, (7) Akt-Ser473, (8) AMPKα-Thr172, (9) S6 Ribosomal Protein-Ser235/236, (10) mTOR-Ser2448, (11) HSP27-Ser78, (12) Bad-Ser112, (13) p70S6 Kinase-Thr389, (14) PRAS40-Thr246, (15) p53-Ser15, (16) p38-Thr180/Tyr182, (17) SAPK/JNK-Thr183/Tyr185, (18) PARP-Asp214, (19) caspase-3–Asp175, (20) GSK-3β-Ser9. Proteins that showed differences in expression/phosphorylation levels compared to the control protein extract are indicated. **(C,D)** Determination of signal intensity of spots from microarrays (**A,B**, respectively), was conducted by densitometry using ImageJ software (NIH). Data represent the means ± SE of at two independent assays with spot duplication for each antibody, using protein extracts obtained from at least three tumors of each mouse group. Bars represent means ± SE of four densitometry determinations. One-way ANOVA was carried out, with Bonferroni *post-hoc* test; statistical significance (*P* < 0.05) of differences between treatments is indicated with lowercase letters. Control, healthy mice; Tumor (T), mice with tumor implantation without treatment; T + CDPs-t8, mice with tumor implantation treated with CDPs at the eighth day of melanoma implantation.

To identify elements dysregulated in cancer and possible cell-signaling pathways targeted by the antitumorigenic effect of the CDPs in the xenografted melanoma mouse model, protein extracts obtained from tumor tissue were used for an immunodetection approach. Results of the cancer and signaling antibody arrays showed that tumors presented a significant increase in the expression of proliferative cell nuclear antigen (PCNA), p27Kip1, N-cadherin, HIF-1α, Stat-3, Akt-S473, AMPka, mTOR-S2448, HSP27, Bad, PRAS40, SAPK/JNK, and caspase-3 (Figures 5A–C), which were significantly recovered/diminished in their expression/phosphorylation level by CDP treatment. Additionally, in the T + CDPs mouse group, CDPs caused an increment in the expression of elements such as survivin, Met, and EGF (Figures 5A–C).

**Figure 6:**
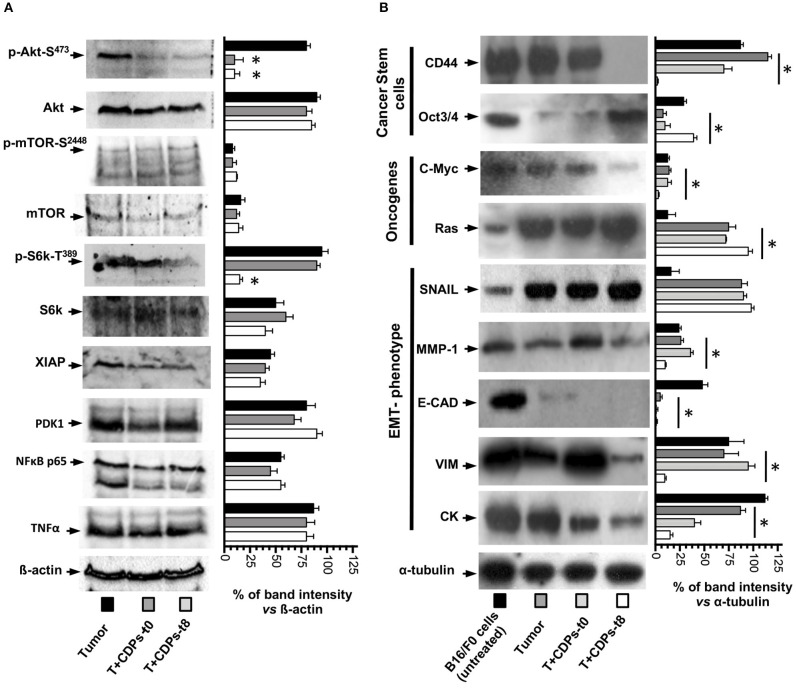
Effects of CDPs on the levels of proteins involved in signaling pathways promoting mouse melanoma growth. Tumor tissue was disrupted, and cell-free protein extracts were used for Western blot assays as described in Materials and Methods. Images correspond to a representative Western blot assay (*n* = 3) using protein extracts of at least three tumors homogenized from the mentioned mouse groups, using the indicated antibodies. **(A)** Antibodies recognized p-Akt-S473, Akt, p-mTOR-S2448, mTOR, pS6K-T389, S6K, XIAP, PDK1, NFκB p65, TNF-α, and β-actin. **(B)** Antibodies used were against CD44, Oct3/4, C-Myc, Ras, SNAIL, MMP-1, E-CAD, VIM, CK-1, and α-tubulin. On the right, graphs correspond to determination of the band intensity from the Western blot assay (left), analyzed by densitometry using the ImageJ software. Data represent the means ± SE of densitometry determinations, *n* = 3 for each antibody, using protein extracts obtained from tumors of each group vs. load control (β-actin or α-tubulin). One-way ANOVA with Bonferroni *post-hoc* test was used to compare treatments with respect to Tumor group. Significant differences (*P* < 0.05) vs. tumor group are indicated with asterisk (*). B16-F0 mouse cells line untreated; (Tumor) mice with tumor implantation without treatment; (T + CDPs-t0) mice with tumor implantation and CDP treatment immediately after injection; (T + CDPs-t8) mice with tumor implantation and CDP treatment at the eighth day of melanoma implantation.

Because we observed an increment in expression of signaling pathways such as PI3k/Akt/mTOR expression in tumors and its recovery level of expression after CDP-treatment, we looked at the activation of Akt-S473, mTOR-S2448, S6k-T389, and other members of signaling pathways such as Xiap, PDK1, NFκB p65, and TNF-α/FasL (Figure 6A). Phosphorylation of Akt-S473 and S6k-T389 was strongly activated in the tumor mouse group (T), whereas a decrease was observed with CDP treatment, but no difference in activation was observed for mTOR-S2448 (Figure 6A). In contrast, Xiap, PDK1, NFκB p65, and TNF-α/FasL did not show significant differences in expression level in the CDP-treated mouse group with respect to the T group (Figure 6A).

We also looked at markers of malignancy (i.e., cancer stem cells, pluripotency, and metastasis). Figure 6B shows that the expression of stem cell marker CD44 was decreased to <30% in protein extracts of tumors from T + CDPs-t0 mouse group and was totally depressed in the T + CDPs-t8 group; in contrast, the Oct3/4 showed low expression levels in the T mice group, with expression levels increased in the T + CDPs-t8 group. With respect to the oncogenes, c-Myc was decreased, but Ras was increased in the T + CDPs mouse group. Furthermore, the expression of EMT markers such as SNAIL increased in tumors and CDP-treated tumors. However, MMP-1, E-cadherin, vimentin, and CK-1 showed a significantly decreased expression in tumors treated with CDPs (Figure 6B). These results clearly indicate that, in addition to the PI3k/Akt/mTOR pathway, other signaling pathways are involved in melanoma development as in B16-F0 cells line as in tumor of xenografted melanoma cells, and these also may be targeted by the bacterial CDPs.

## Discussion

The quest for novel molecules to target cancer has led investigations to look at microbial metabolites. Cyclic peptides constitute a diverse family of molecules mainly of microbial origin that have antimicrobial, immunomodulator, antioxidant, or anticancerigenic activities (7, 21). Recently, CDPs have attracted attention because of their antiproliferative effects on cancer cell lines (3–6, 22). We recently reported that a mixture of CDPs composed of cyclo(L-Pro-L-Tyr), cyclo(L-Pro-L-Val), and cyclo(L-Pro-L-Phe) isolated from *P. aeruginosa* PAO1 culture inhibited the proliferation of HeLa and CaCo-2 cells (4). In addition, we found that bacterial CDPs are stronger antiproliferative agents compared to chemically synthetized analogs (6). Thus, it appears that CDPs from biological sources are more potent than synthetic CDPs due probably to structural differences in stereospecificity.

Cyclodipeptides affected the viability of B16-F0 cells by inducing apoptosis in cultured cells (Figure 1). Although CDP's biological properties have been studied *in vitro*, we now show that CDPs can inhibit melanoma tumor progression in mice. Interestingly, we showed that mice implanted with B16-F0 cells and simultaneously treated with CDPs did not develop tumors, unlike untreated mice. Importantly, in tumors already developed, tumor size was decreased in mice treated with CDPs (Figures 2–4). Mice that developed tumors showed diminished blood cell counts, which correlated with an increment in spleen size (Figures 2, 3), symptoms that are indicative of an anemic status seen in patients with cancer (23). Cyclodipeptide treatment of the tumor-induced mice resulted in a recovery of hematologic parameters (Figures 3A–C). Furthermore, LDH, ALT, and AST increased in mice with tumors as described in liver and breast cancers (24, 25), but the levels of these enzymes significantly decreased when mice were treated with CDPs (Figure 3D). Interestingly, the control mouse group treated with CDPs did not show altered values of these blood and enzymes levels, suggesting that, at least, the injected amount of CDPs was not toxic to mice. Levels of LDH, ALT, and AST are used as markers of disease evolution of anticancer treatments (25). We observed a recovery of the levels of LDH and AST in mice treated with CDPs similar to those found in healthy mice (Figure 3D). This was not observed in the untreated group. These results indicate an improvement in health of mice bearing tumors treated with CDPs. In addition, histological and immunohistochemical data showed strong nuclear fragmentation and decreased Bcl-2 and Ki67 cancer marker levels in tumor tissues from mice treated with CDPs (Figure 3E). Images of tissue showed an exacerbated generation of ROS in tumors from CDP-treated mice, confirming the induction of apoptosis, dependent on mitochondrial dysfunction and cytochrome *c* release.

An important issue to consider in therapeutic treatments is the drug bioavailability; in this sense, we improved the bioavailability of the CDPs using a suspension in β-cyclodextrins. Results obtained in our xenografted melanoma mouse model showed that the CDPs dissolved in cyclodextrins were more efficient in inhibiting tumorigenesis than those dissolved in DMSO (Figure 4), suggesting that the antitumorigenic effect of the CDPs could be improved by utilization of an appropriate drug vehicle.

To determine the molecular mechanism involved in the antitumorigenic effect of CDPs in the xenografted melanoma mouse model, we utilized antibody arrays to screen in tumors, differential expression of proteomic elements related to cancer disease, and intracellular signaling pathways (Figure 5). Previously, we found that the CDP mixture from *P. aeruginosa* PAO1 was able to repress phosphorylation of both Akt-S473 and S6k-T-389 protein kinases in HeLa cells at short treatment times (6). The PI3K/Akt/mTOR pathway is up-regulated in more than 70% of cancer types (26, 27). In our murine melanoma model, we also found up-regulation of PI3k/Akt/mTOR pathway (Figure 6). In this study, also hyperactivation of phosphorylation in melanoma tumors from mice was found, where the level of phosphorylation was decreased in tumors from CDP-treated mice. Thus, it further confirms that CDPs targeted the mTOR pathway, which is critical for cell growth and proliferation. Apoptosis dependent on the Akt-Ser473 inhibition and downstream target proteins by CDPs in our melanoma model indicates the participation of the mTORC1 and mTORC2 complexes in blocking the PI3K/Akt/mTOR signaling pathway; in agree with recent findings described in HeLa cells (28). In agreement with these results, dual inhibition of the mTORC1 and mTORC2 signaling pathways has been proposed as effective therapeutic targets in neoplasias (29, 30). It suggests that CDPs can be considered as potential therapeutic compounds in melanoma by causing dual inhibition of mTORC1 and mTORC2 complexes.

Additionally, we observed an up-regulation of HIF-1α protein in tumors of xenografted melanoma mice (Figure 5). The HIF-1 suppressor is a master regulator of elements involved in glycolysis and is dysregulated in tumorigenesis and invasiveness. It is well-known that the regulation of HIF-1 is closely related to the PI3K/Akt/mTOR pathway. Functionally, it has been shown that Akt and HIF-1 interact synergistically during the development of melanoma (31). Our data show that the PI3K/Akt/mTOR and HIF-1 cross-talk pathways are implicated in mouse melanoma development and that CDPs targeted these pathways. Because HIF-1 regulates processes such as survival, apoptosis, glucose metabolism, angiogenesis, and invasiveness by inducing EMT regulators (32–34), it is attractive to postulate that the antitumor effects of CDPs may be mediated by affecting HIF-1 and reflected in the blocking of cell invasion. In addition, the PCNA, which acts during the S and G2 phases of the cell cycle, is considered a marker of cell proliferation, which actively participates in a number of signaling pathways responsible for cell survival (35).

We found that the transcription factor SNAIL is up-regulated in melanoma tumors, whereas expression of CD44 and E-Cad is down-regulated (Figure 6B). SNAIL is a prominent inducer of EMT and strongly represses E-cadherin expression. Cyclodipeptides increased SNAIL levels and decreased levels of the hyaluronan receptor CD44, a cell surface adhesion receptor that is highly expressed in many cancers and regulates metastasis by alternative splicing and recruitment of CD44 to the cell surface (36).

Furthermore, important tumorigenic markers were repressed in the CDP-treated mice, such as C-Myc, MMP-1, E-cadherin, vimentin, and CK-1 (Figure 6B). These data support the hypothesis that CDPs may promote the activation/repression of the main components of the EMT pathway to induce apoptosis in tumors and probably inhibit tumorigenesis and invasiveness. On the other hand, Xiap, PDK1, NFκB p65, and TNF-α/FasL did not show significant differences in their expression level in the CDP-treated mouse group with respect to the T group (Figure 6A), suggesting that these elements play important roles in signaling pathways in which their participation is not affected by the CDPs; however, this does not rule out their involvement.

**Figure 7:**
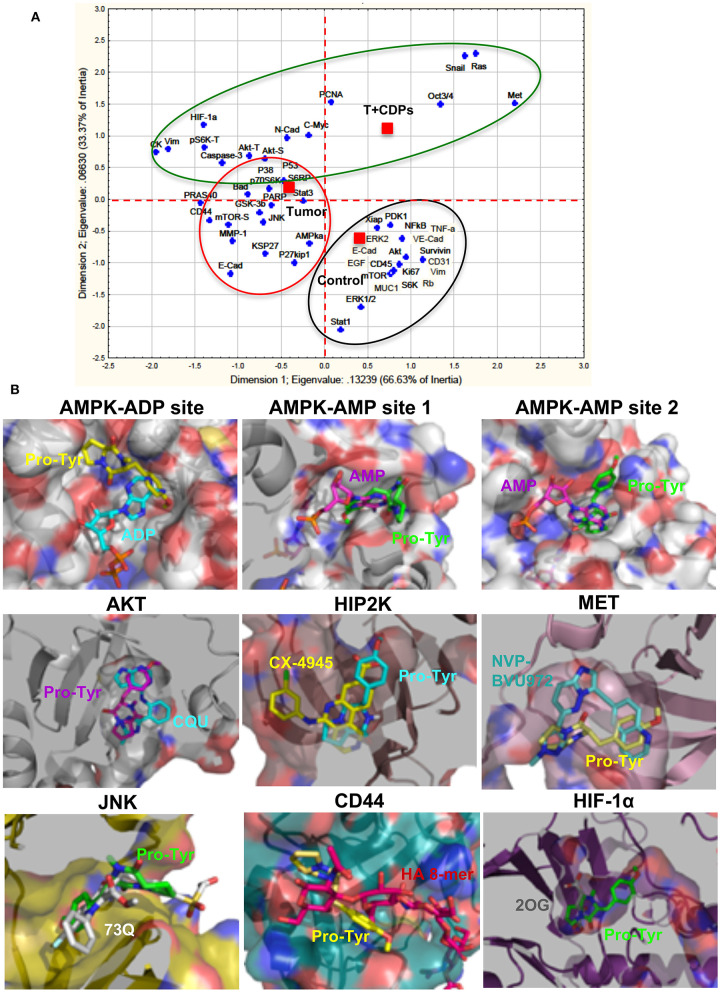
Correlation analysis of proteomic approach on xenografted tumor melanoma B16-F0 cells in mice treated with CDPs and docking analysis. **(A)** Correlation analysis of densitometry data from microarrays and Western blots (Figures 5, 6) were analyzed by multivariate exploratory techniques, using the correspondence analysis (CA) of the STATISTICA Software System 8.0 (Stat Soft Inc., Tulsa, OK, USA). Correlation groups are as follows: healthy mice (control); mice with tumor implantation without treatment (Tumor); mice with tumor implantation and CDP treatment (T + CDPs). **(B)** Docking analysis of the CDP cyclo(L-Pro-L-Tyr) in the binding sites of tyrosine-kinases. A slice of the protein showing the catalytic or inhibitor binding sites of protein kinases is presented with the orientation of the CDP (Pro-Tyr) in the predicted conformations. The protein is shown as surface with the interior in cartoon; Pro-Tyr and other molecules are shown as sticks colored by elements. Pro-Tyr, cyclo(L-Pro-L-Tyr); CQU, (N-[2-(5-methyl-4H-1,2,4-triazol-3-yl)phenyl]-7H-pyrrolo[2,3-d]pyrimidin-4-amine); CX-4945, 5-[(3-chlorophenyl)amino]benzo[c] [2,6]naphthyridine-8-carboxylic acid; NVP-BVU972, 6-[[6-(1-methylpyrazol-4-yl)imidazo[1,2-b]pyridazin-3-yl]methyl]quinolone; 73Q, 6-chlor-9-hydroxy-1,3-dimethyl-1,9-dihydro-4H-pyrazolo[3,4-B]quinolin-4-one; HA 8-mer, hyaluronan octamer; 2OG, 2-oxoglutarate. Models were carried out with PYMOL 2.1.0 (The PyMOL Molecular Graphics System, version 2.1.0; Schrödinger, LLC, New York, NY, USA).

An approach analysis of the multiple factors and pathways evaluated in our melanoma tumorigenesis model with treatment with bacterial CDPs was conducted using a statistical correspondence analysis (Figure 7A). Data show that the cancer factors clearly correlated in up-expressed/activated and down-expressed/inactivated as widely described (Figure 7A). An important number of proteins are associated with the response variable (control), which were not modified in their expression level in tumors CDPs-treated and untreated groups. A second group of proteins that showed significant changes in their expression levels in the proteomic approach was associated with the response variable Tumor mouse group which developed tumors without treatment. This group of proteins corresponds to oncogenes and tumor suppressors widely associated with tumorigenesis. Finally, a third group of proteins highly implicated in the control of tumorigenesis, invasiveness, and signaling pathways such as PI3K/Akt/mTOR, Ras/ERK, EMT, CSC, and so on, was associated with the CDPs-treated mouse group (Figure 7A). From this correlation analysis, some representative proteins of each group were selected and analyzed by molecular docking using the crystallographic structures with ligands, such as substrate or inhibitors, and evaluated for the feasibility of interaction with the CDPs as modulator molecules (Figure 7B). Docking approach showed that the CDPs may interact with protein kinase members of multiple signaling pathways. The cyclo(L-Pro-L-Tyr) and cyclo(L-Pro-L-Phe) showed better predicted interaction values (*Ki* of 2–65 μM and binding energy of −7.8 to −7 kCal/mol), than cyclo(L-Pro-L-Val) (Table S1). Docking revealed that the CDPs have the potential to interact in different sites of protein kinases such as substrate-binding site, inhibitor-binding site or co–substrate-binding site. Analysis also predicted a differential affinity for the CDPs, being cyclo(L-Pro-L-Tyr) in general the most potent as inhibitor. Structural images of proteins kinases AKT, HIPK2, AMPK, MET, JNK, HIF-1α, and CD44 with the interaction with the cyclo(L-Pro-Tyr) are shown in Figure 7B. In agreement with our results, some reports have described that some melanoma treatments implicate the use of tyrosine kinase inhibitors such as the MAPK pathway, PI3K (16). Thus, we can propose that inhibition or activation of the protein kinases such as AKT, HIPK2, AMPK, MET, JNK, HIF-1α, and CD44 by treatment with CDPs in the mouse melanoma model could involve the blocking of the substrate binding site as a molecular mechanism.

Melanoma tumors in mouse showed a significant decrease in factors involved in survival, proliferation, invasiveness, angiogenesis, and glucose metabolism. Thus, our data suggest that bacterial CDPs suppress the activation of the signaling pathways associated with the onset of tumorigenesis governed by PI3K/Akt/mTOR, Ras-ERK, PI3K/JNK/PKA, p27Kip1/CDK1/survivin, MAPK, HIF-1, EMT, and CSC not actually shown. Although the mode of action of CDPs is not totally elucidated, we propose that CDPs target multiple pathways and processes (oncogenes and tumor suppressors) involved in tumor formation and progression by blocking the catalytic site conserved in the serine–threonine kinase family. Findings indicate that the multiple signaling pathways are implicated in melanoma aggressiveness, showing that bacterial CDPs interfere during melanoma development by a mechanism dependent on protein kinase inhibition. Our data suggest that CDPs have potential antiproliferative properties to be used as antineoplastic drugs.

## Data Availability Statement

All datasets generated for this study are included in the article/Supplementary Material.

## Ethics Statement

The animal study was reviewed and approved by the Institutional Committee for Use of Animals of the Universidad Michoacana de San Nicolás de Hidalgo. The study was carried out in accordance with the Mexican Official Regulations for the Use and Care of Animals (NOM 062-ZOO- 1999, Ministry of Agriculture, Mexico).

## Author Contributions

JC-G: conception and design. MD-M, LH-P, JG-P, AD-P, and LM-A: development of methodology. JC-G, JR-Z, GP-R, and JM: analysis and interpretation of data. JC-G, JR-Z, HR, GP-R, and JM: writing, review, and/or revision of the manuscript. JC-G, JR-Z, and HR: administrative, technical, or material support. JC-G, JR-Z, and GP-R: study supervision. All authors read and approved the final manuscript.

## Conflict of Interest

The authors declare that the research was conducted in the absence of any commercial or financial relationships that could be construed as a potential conflict of interest.
