# Supplementary material for: Bacterial Cyclodipeptides Target Signal Pathways Involved in Malignant Melanoma
Source: Front Oncol. 2020 Jul 24;10:1111. doi: 10.3389/fonc.2020.01111 (PMC7393205; doi:10.3389/fonc.2020.01111)
Supplement: Supplementary file 1 [file Table_1.docx]

Table S1. Results of docking of the bacterial cyclodipeptides on the binding sites of some signal pathways components.

|  | **Cyclo(L-Pro-L-Tyr)** | | **Cyclo(L-Pro-L-Phe)** | | **Cyclo(L-Pro-L-Val)** | |
| --- | --- | --- | --- | --- | --- | --- |
|  | **B. E.** (kCal/mol) | ***Ki***  (µM) | **B. E.**  (kCal/mol) | ***Ki***  (µM) | **B. E.** (kCal/mol) | ***Ki***  (µM) |
| **AKT (3CQU)** |  |  |  |  |  |  |
| Inhibitor site | - 7.78 | 2.0 | -7.66 | 2.45 | -6.35 | 22.03 |
| Protein binding site | -5.43 | 104.3 | -5.15 | 167.4 | -4.74 | 338.1 |
|  |  |  |  |  |  |  |
| **HIPK2 (6P5S)** |  |  |  |  |  |  |
| Inhibitor site | -7.58 | 2.78 | -7.12 | 6.03 | -5.7 | 65.89 |
|  |  |  |  |  |  |  |
| **AMPK (5UFU)** |  |  |  |  |  |  |
| ADP binding site | -6.78 | 10.75 | -6.76 | 11.01 | -5.77 | 59.27 |
| AMP1 binding site | -6.57 | 15.19 | -6.03 | 38.26 | -5.8 | 56.32 |
| AMP2  binding site | -6.2 | 28.71 | -5.53 | 88.57 | -4.84 | 228.4 |
|  |  |  |  |  |  |  |
| **MET (3QTI)** |  |  |  |  |  |  |
| Inhibitor site | -6.73 | 11.61 | -7.16 | 5.67 | -5.97 | 41.73 |
|  |  |  |  |  |  |  |
| **JNK (2G01)** |  |  |  |  |  |  |
| Inhibitor site | -6.38 | 21.24 | -6.65 | 13.27 | -5.76 | 60.11 |
| Protein binding site | -4.97 | 228.3 | -3.98 | 1200 | -4.67 | 374.4 |
|  |  |  |  |  |  |  |
| **HIF-1α (5JWP)** |  |  |  |  |  |  |
| 2-Oxoglutatate binding site | -5.75 | 60.68 | -6.13 | 31.95 | -5.63 | 75.07 |
|  |  |  |  |  |  |  |
| **CD44 (2JCR)** |  |  |  |  |  |  |
| Hyaluronan 8-mer binding site | -5.71 | 65.63 | -5.36 | 117.6 | -4.4 | 593.7 |

B.E., binding energy ; *Ki*, inhibition constant.
